# Guiding Lights in Genome Editing for Inherited Retinal Disorders: Implications for Gene and Cell Therapy

**DOI:** 10.1155/2018/5056279

**Published:** 2018-05-08

**Authors:** Carla Sanjurjo-Soriano, Vasiliki Kalatzis

**Affiliations:** ^1^Inserm U1051, Institute for Neurosciences of Montpellier, Montpellier, France; ^2^University of Montpellier, Montpellier, France

## Abstract

Inherited retinal dystrophies (IRDs) are a leading cause of visual impairment in the developing world. These conditions present an irreversible dysfunction or loss of neural retinal cells, which significantly impacts quality of life. Due to the anatomical accessibility and immunoprivileged status of the eye, ophthalmological research has been at the forefront of innovative and advanced gene- and cell-based therapies, both of which represent great potential as therapeutic treatments for IRD patients. However, due to a genetic and clinical heterogeneity, certain IRDs are not candidates for these approaches. New advances in the field of genome editing using Clustered Regularly Interspaced Short Palindromic Repeats (CRISPR) and CRISPR-associated protein (Cas) have provided an accurate and efficient way to edit the human genome and represent an appealing alternative for treating IRDs. We provide a brief update on current gene augmentation therapies for retinal dystrophies. Furthermore, we discuss recent advances in the field of genome editing and stem cell technologies, which together enable precise and personalized therapies for patients. Lastly, we highlight current technological limitations and barriers that need to be overcome before this technology can become a viable treatment option for patients.

## 1. Introduction

The eye, and more specifically the retina, as an extension of the central nervous system (CNS), provides a powerful and unique “window” to study neuronal diseases. The retina shares anatomical and developmental characteristics with the brain [1]. For example, it is relatively immunoprivileged and has specialized immune responses similar to the ones found in the brain and spinal cord [2, 3]. In addition, it is surrounded by the inner blood-retinal barrier (BRB), which is composed of the same nonfenestrated endothelial cells as those found in the blood-brain barrier (BBB) [4]. Due to the accessibility of the eye by modern techniques of vitreoretinal surgery, it is not surprising that major research and understanding in the context of the CNS has emerged from studies of the retina and the optic nerve [5–11]. Furthermore, the significant compartmentalization of the eye, and specifically the retina, has allowed it to become a prototype for the development of innovative therapies and has brought ocular diseases to the forefront of clinical translation for gene- and cell-based therapies. Here, we will specifically review current progress in these therapeutic strategies for diseases of the posterior retina (namely the neuronal photoreceptor cells). Optic neuropathies affecting the anterior retina (retinal ganglion cells (RGCs)) and optic nerve are beyond the scope of this review.

## 2. The Retina

**Figure 1:**
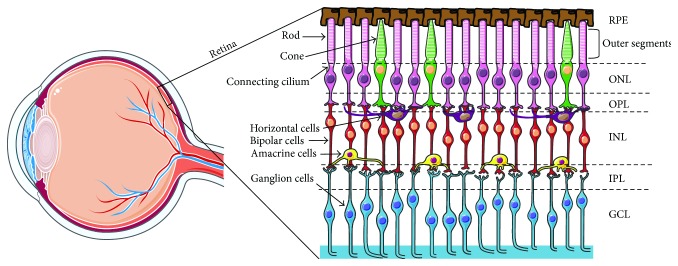
Schematic representation of the retina and the retinal cell layers. The retina is a layered structure lining the back of the eye consisting of a pigmented layer, the RPE, and a multilayered neuroretina. The RPE is in close contact with the outer segments of the photosensitive rod and cone cells of the neuroretina. The connecting cilium connects the photoreceptor outer segments with the cell bodies, which constitute a layer known as the outer nuclear layer (ONL). The axons of the photoreceptors synapse with the neuronal (bipolar, amacrine, and horizontal) cells of the inner nuclear layer (INL) via the outer plexiform layer (OPL). The axons of the INL cells in turn synapse with the ganglion cell layer (GCL) via the inner plexiform layer (IPL). The axons of the ganglion cells converge to form the optic nerve.

The retina is an embryonic extension of the prosencephalon [12]. It lines the back of the eye and consists of multiple cell layers that are responsible for the detection and processing of visual information. The retina has a highly structured architecture that can be divided into a posterior pigmented monolayer and an anterior multilayered neuroretina. The posterior layer, the retinal pigment epithelium (RPE), plays an important role in protection (excess light absorption, phagocytosis, water and ion transport) and support (growth factor section, nutrient transport) of the photoreceptor layer [13, 14]. The neuroretina is highly stratified, and it is composed of three layers of specialized neurons that are interconnected by two synaptic layers (Figure 1). The first layer comprises the photosensitive rod and cone photoreceptor cells with their characteristic outer segments, within which the phototransduction process that follows light interaction takes place. Light intensity dictates which photoreceptor cells are used. In bright light, it is the centrally prevalent cones, and in low light, it is the peripherally prevalent rods. The photoreceptors then synapse with interneurons within the second layer, which transmit the electrical signal arriving from the photoreceptors to the RGCs in the third layer [15]. The axons of the RGCs form a nerve fibre layer, which becomes the optic nerve, and hence, the signal is transmitted from the eye to the brain for image interpretation. The inability to convert the light signal and transmit the electrical signal to the brain is the primary cause of visual impairment in the developing world. A large proportion of cases is due to dysfunction and/or loss of photoreceptors caused by a series of risk factors including age, diabetes, and genetics [16]. The latter gives rise to a specific subset of conditions referred to as inherited retinal dystrophies.

## 3. Inherited Retinal Dystrophies

Inherited retinal dystrophies (IRDs) are a genetically and clinically heterogeneous group of neurodegenerative disorders that lead to progressive visual impairment [16, 17]. They affect approximately 1 in 2000 individuals worldwide [18]. IRDs have been associated with mutations in more than 250 genes (see http://www.sph.uth.tmc.edu/Retnet), affecting the development, function and/or survival of the photoreceptors, and RPE [19], and with autosomal dominant, recessive, or X-linked transmission [16]. Furthermore, complex, multifactorial, and heterogeneous diseases such as age-related macular degeneration (AMD) are also considered retinal dystrophies.

IRDs can be divided into nonsyndromic forms, characterized by an isolated retinal phenotype, or syndromic forms, in which another organ in addition to the eye is affected. Nonsyndromic IRDs can be further broken down into subgroups based on the disease progression and the region of the retina that is affected. Firstly, progressive conditions affecting exclusively the central retina (macula), leading to central vision loss, are known as macular dystrophies. The most common example is Stargardt disease with a prevalence of 1/10000, which is due to mutations in the gene *ABCA4* [20]. Secondly, progressive conditions affecting the retina more widely can be classified depending on the type of photoreceptor that degenerates initially. Rod-cone dystrophies, where the rods are first affected, are characterized initially by night blindness and subsequently by peripheral vision loss; the most prevalent example (1/4000) is retinitis pigmentosa (RP), caused by mutations in over 80 genes [21]. By contrast, in cone-rod dystrophies, the cones are first affected, leading to decreased sharpness of visual acuity and blind spots in the center of the visual field; *ABCA4* mutations also account for the majority of these cases [22].

When both the macula and the peripheral retina are affected and there is a rapid retinal degeneration from birth, the condition is known as Leber congenital amaurosis (LCA; prevalence of 1/50000), of which 18 types are recognized. In addition, if the retinal changes are associated with a degeneration of the choroid, a highly vascular, pigmented tissue underlying the retina, these diseases are referred to as chorioretinopathies. Choroideremia (CHM) is the most common example (prevalence of 1/50000) in this group. The most common form of syndromic IRD is the heterogeneous Usher syndrome group (prevalence of 1/20000), which is characterized by RP and hearing loss [23]. Usher syndrome is further subdivided into three subtypes depending on the severity and progression of the hearing loss and the age of onset of the RP. Usher syndrome type 1 (USH1) is the most severe; Usher syndrome type 2 (USH2) is the most common presenting moderate to severe symptoms. Lastly, Usher syndrome type 3 (USH3) presents a moderate phenotype and variable progression and onset of the disease.

The monogenic nature of IRDs coupled to the accessibility and immunoprivileged nature of the human eye has led to the advancement of pioneer gene therapies that hold promise for the development of future treatments. Most predominantly, IRDs have been targets for gene augmentation therapy [24]. More recently, gene correction of the causative gene, either by inactivation of the autosomal dominant allele or by correction of the recessive or X-linked alleles, has been explored as a possible treatment strategy. Currently, there is no standardized therapeutic option in the clinic for IRDs, due to the challenges of a diverse genetic landscape, fluctuating disease prevalence, variable age of onset and clinical course, and the specificity of the therapeutic products.

## 4. Gene Augmentation Therapy for IRDs

Gene augmentation therapy provides a normal copy of a mutated gene into native cells and hence is applicable for the treatment of haploinsufficiency or loss-of-function mutations. Most commonly, but not exclusively, the genes are vehicled by viral vectors, a pertinent example being adeno-associated viral (AAV) vectors [25]. AAV vectors present specific characteristics such as low immunogenicity and toxicity, lack of pathogenicity, long-term transgene expression, and relative ease in manipulating genetic elements, making them the safest and most effective viral vector platform for gene delivery into the retina to date [26]. Delivery can be achieved by subretinal injection, where the vector is administered into the subretinal space between the photoreceptors and RPE, which can result in the transduction of both cell types depending on the serotype used [27]. Other methods, such as intravitreal delivery, are less invasive and thus result in fewer complications postsurgery, but the delivery of the therapeutic genes, particularly to the posterior retina, is less effective [28].

A major milestone in gene augmentation therapy for IRDs was achieved in 2001 using a canine model for LCA2 due to mutations in the gene *RPE65 (RPE65^−/−^)*. AAV2/2-mediated delivery of *RPE65* led to the long-term restoration of vision in treated dogs [29]. Following this study, multiple phase 1/2 clinical gene therapy trials assessed the effects of subretinal administration of AAV-RPE65 and demonstrated improved vision in some patients with no adverse effects of the vector [30–34]. A phase 3 clinical trial for LCA2, in which the therapeutic vector was administrated in both eyes, was subsequently launched. The vision of the treated group significantly increased compared to the control group, and this became the first ocular clinical trial in which both eyes were treated successfully [35]. As a consequence, the corresponding vector has been recently commercialized as a drug under the name of voretigene neparvovec (Luxturna).

Hot on the heels of the AAV-RPE65 trial, phase 1/2 clinical trials for the X-linked chorioretinopathy choroideremia were initiated [36, 37] following preclinical studies in *Chm^null/WT^* mice [38]. These trials are also using an AAV2/2 vector, administered subretinally, to vehicle the causative *CHM* gene into both photoreceptors and the RPE. However, preclinical studies have shown that other AAV serotypes such as AAV2/5 [39] and AAV2/8 [40] are also effective for choroideremia. Lastly, a phase 1 clinical trial to treat RP was performed using an AAV2/2 vector to vehicle the causative gene *MERTK* [41] confirming the safety profile of this vector serotype. A variety of other clinical trials have been initiated worldwide for other IRD genes using alternative AAV serotypes, but the results are still forthcoming.

Despite its numerous advantages, AAV vectors are limited by their cloning capacity (<4.7 kb) [42–44]. To overcome this limitation, efforts have turned to the use of equine infectious anemia virus- (EIAV-) based lentiviral vectors, which although integrative are nonpathogenic to humans. An EIAV vector was first tested in the case of the *ABCA4* gene, which has a 6.8 kb coding sequence. Preclinical studies in the mouse *Abca4^−/−^* model showed a reduction in toxic A2E accumulation in the RPE of treated mice as compared to controls [45]. Following biodistribution and safety studies of the corresponding EIAV ABCA4-carrying vector [46], a clinical trial was begun in 2011, but the results are still pending. Similarly, an EIAV vector, carrying the *MYO7A* gene (6.5 kb), was tested for its efficiency in the treatment of RP associated with Usher syndrome 1B [47]. Proof-of-concept studies in the mouse *Myo7A^−/−^* model suggested that the vector was able to prevent light-induced retinal degeneration [48]; however, the results of the clinical trial begun in 2012 are also pending. The outcome of these two EIAV clinical trials is essential to assess the suitability of lentiviral-based vectors for therapy of IRDs due to large causative genes.

Since the landmark canine LCA2 study by Acland et al., the progress in precision medicine research has continued to develop. However, several challenges remain to be overcome. Despite variations in visual improvements among treated patients in the LCA2 trials, long-term follow-up studies showed that the retinal structure continued to degenerate [49, 50]. This could be attributed to the advanced disease course at the time of treatment at which point the degeneration process could no longer be halted [51–53]. Advanced stages of retinal degeneration are incompatible with gene augmentation therapy, which, to be successful, requires that the nonfunctional target cells are still alive. Such patients might benefit better from cell-based transplantation therapy, which has the potential to restore visual function as detailed later.

An alternative explanation for the continual degeneration posttreatment could be inefficient vector transduction [52, 54]. Achieving correct levels of gene expression is essential for a robust and significant rescue of the phenotype [50, 55, 56]. This may be improved by the use of alternative [39, 57, 58] or modified [59] AAV serotypes, which have been shown to have a higher transduction efficiency than AAV2/2 in multiple species, or optimized promoter and/or codon-optimized cDNA sequences, which can stabilize transcript expression and hence increase protein levels [60, 61]. Finally, gene augmentation strategies are not convenient approaches for treating dominant or dominant-negative mutations, in which the mutated allele causing the disease needs to first be inactivated so that it does not interfere with the wild-type copy [62]. This is generally most easily accomplished by dual (wild-type and mutant) allele silencing prior to gene augmentation [63–66]. Therefore, despite the limited benefit demonstrated in clinical trials using AAV-mediated retinal gene augmentation therapy for the treatment of recessive mutations, other approaches for treating IRDs are being investigated with promising results.

## 5. Genome Editing for the Treatment of IRDs

**Figure 2:**
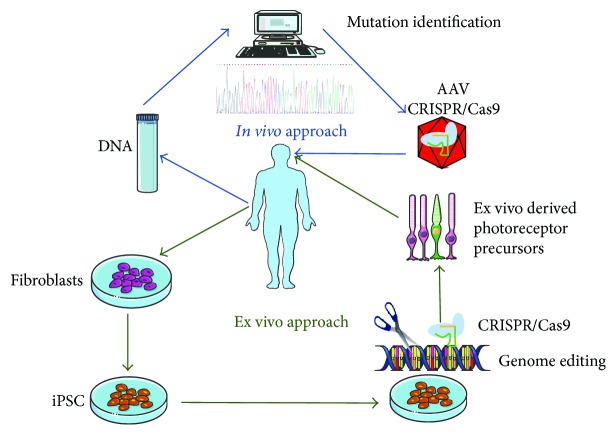
Therapeutic approaches for treating retinal dystrophies. For an *in vivo* approach (indicated in blue), the patient's DNA is isolated, and genetic screening is carried out to identify the pathogenic mutation causing the retinal phenotype. Delivery of the CRISPR/Cas9 components to correct the pathogenic mutation *in vivo* is achieved via AAV vectors administrated directly to the retina of the patients. For an ex vivo approach (in green), patient's fibroblasts with a known mutation in an IRD gene are isolated and reprogrammed to patient-specific iPSC. Genome editing of iPSCs is carried out using the CRISPR/Cas9 system. The corrected iPSCs are further differentiated into retinal cells, which can then be reimplanted into the patient's retina.

Providing a wild-type copy of the mutated allele to restore a phenotype does not directly impact the pathogenic host gene. In contrast, a genome-editing approach has the potential of correcting the mutation directly in the patient's DNA. This approach could thus fill the void left by gene augmentation therapy in the case of large causative genes or dominant mutations. There are potentially two different approaches in the case of genome editing: an *in vivo* approach whereby the disease-causing mutations are corrected directly in the retina and an ex vivo approach in which the mutation is corrected in the patient's cells in view of future cell transplantation (Figure 2). The advances and current progress for both strategies will be summarized here. In addition to correcting pathogenic mutations, genome editing has also been used in a variety of preclinical models to further understand disease pathogenesis and to determine feasible treatment options.

Genome editing has advanced at an exceptionally rapid rate, creating huge impacts on biotechnology and biomedicine. The genome-editing era was initially triggered by the use of engineered meganucleases and zinc finger nucleases (ZFN) to specifically target a genomic sequence. Later, the development of transcription activator-like effector nucleases (TALEN) and more recently, the Clustered Regularly Interspaced Short Palindromic Repeats (CRISPR) and CRISPR-associated genes (Cas) system have led to a scientific genome-editing revolution.

### 5.1. ZFNs and TALENs

**Figure 3:**
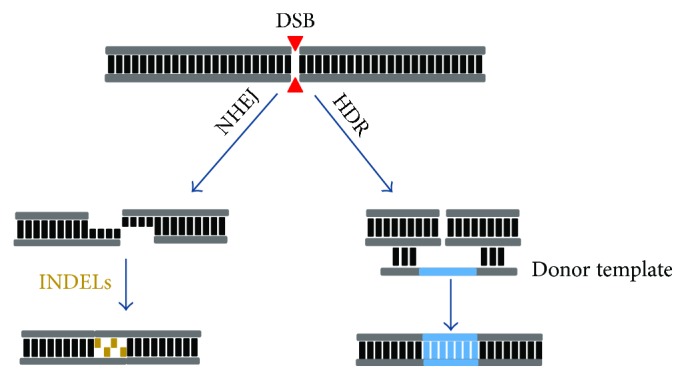
Schematic representation of a double-stranded break (DSB; red arrowheads), which can be repaired through nonhomologous end-joining (NHEJ) or homology-directed repair (HDR) pathways. The introduction of a double-strand break in the DNA will typically undergo the error-prone NHEJ repair pathway, which results in insertions and deletions (INDELs) of variable length that will lead to premature stop codon formation. HDR, an error-free repair pathway, occurs using a wild-type donor template with homology to the target site, which serves as a template for precise gene correction of the host's DNA.

Efficient genome editing, regardless of which tool is used, is based upon the introduction of a double-strand break (DSB) at a precise point in the genome, which rapidly stimulates one of the two DNA repair pathways of the cell [67, 68]. The nonhomologous end-joining (NHEJ) pathway is the default method of repair, introducing insertions and deletions (INDELs) that normally will result in a nonfunctional genetic product [69]. Alternatively, homology-directed repair (HDR) uses the sister chromatids from a homologous chromosome as a template, or, in the case of directed genome editing, a donor template containing the desired sequence [70] (Figure 3). HDR occurs much less frequently than NHEJ, since homologous recombination naturally occurs in the late S and G2 phases of cellular division [71].

**Figure 4:**
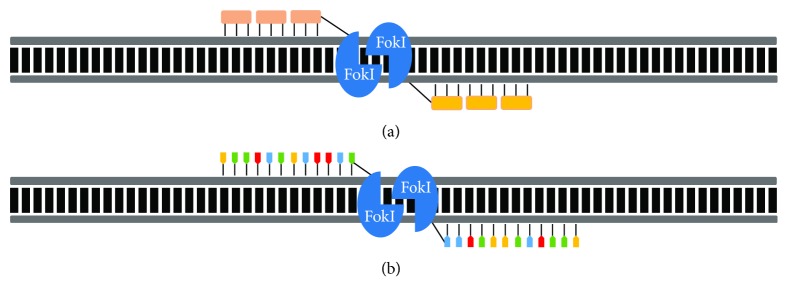
Schematic representation of the structure of a zinc finger nuclease (ZFN) and transcription activator-like effector nucleases (TALENs). (a) Cartoon of a ZFN dimer bound to DNA. ZFNs consist of two functional domains. A DNA-binding domain composed of three zinc finger modules, each one recognizing a unique triplet (3 bp) in the DNA. The DNA-cleaving domain composed of the FokI nuclease is attached to the zinc finger modules and induces the DSB in the DNA. (b) Cartoon of a TALEN dimer bound to DNA. TALENS bind DNA using the TAL effector recognizing individual nucleotides forming the DNA-binding domain. In addition, a DNA-cleaving domain comprised of the FokI nuclease is also present and will induce the DSB at the precise location in the DNA.

To induce a DSB, ZFNs and TALENs need to be guided to the target sequence by a protein DNA-binding domain. They therefore rely on the engineering of new proteins for each target, which has made genome editing difficult, laborious, and challenging [72]. Zinc finger proteins are a class of transcription factors that bind DNA through Cys2-His2 zinc finger domains [73]. ZFNs consist of a modifiable zinc finger domain designed to bind and target specific sequences in the genome and a cleavage domain consisting of the FokI nuclease [74, 75]. The cleavage of the DNA at the desired site is triggered by the dimerization of FokI; thus, two sets of ZFN on either side of the cleavage site are needed for the introduction of the DSB [76]. Similarly, TALENs are engineered by fusing a TAL effector DNA-binding domain with a FokI nuclease cleavage domain [77, 78]. TAL proteins are made of tandem repeats binding to individual nucleotides, which is different to ZFNs in which a zinc finger domain can bind to three different nucleotides (Figure 4). TALENs emerged as an alternative to ZFNs, as they represented a quicker turnaround from design to implementation and a more affordable option. Nonetheless, TALENs are relatively large proteins and contain repetitive DNA sequences resulting in TALEN inactivation [79], making genome editing still very challenging for researchers. In addition, similar to ZFNs, engineering of novel proteins for each DNA target is required.

Despite the challenges, ZFNs have been used as a proof-of-concept treatment for retinal disease. Human embryonic retinoblast cells expressing the Pro23His mutation in the Rhodopsin *(RHO)* gene were targeted with ZFNs. An increase in homologous recombination events occurred when the ZFNs were transfected with a homologous donor template compared to delivery of the ZFNs alone [80]. Similarly, researchers achieved site-specific gene correction in HEK293 cells stably expressing a missense mutation in *Ush1c*, causing Usher syndrome 1C. The authors reported correction of the pathogenic mutation by homologous recombination triggered by ZFNs and a donor plasmid template, when both were transfected to the cells [81]. These studies were the first to demonstrate the feasibility of gene targeting for retinal dystrophies using ZFNs. The major limitation for the applicability of ZFN relies on the design of the zinc fingers to bind every combination of three base pairs present in the genome, which has not yet been achieved. Thus, many sites cannot be targeted using these engineered nucleases [77]. TALEN engineering has also been applied to the retina for the correction of a mutation in the *Crb1^rd8^* mouse, a model for LCA8. The mouse oocytes were treated with mRNA-encoding TALENs targeting the *Crb1^rd8^* allele together with a single-stranded oligonucleotide (ssODN) to correct the pathogenic allele. HDR triggered by TALEN and ssODN repair template was observed in 27% of the treated mice embryos, which presented an improvement of the ocular defects [82].

### 5.2. CRISPR/Cas Systems

The CRISPR/Cas system represents a novel and efficient method for genome editing compared to ZFNs and TALENs. CRISPR were first noticed in the bacterial genome in 1987 and described as an “unusual structure” in the 3′ region of the *iap* gene, containing 29-base pair repeats interspaced by 32 nonrepetitive nucleotides [83]. Later, similar repeats were found in numerous bacteria and archaea [84–86]. It was in 2000 when the acronym CRISPR was given to unify these repeats observed in the bacterial genome [87, 88]. In addition, researchers discovered several clusters of protein-coding genes adjacent to these repeats, and they were subsequently called CRISPR-associated genes or Cas genes [87]. Evidence emerged that CRISPR loci might be involved in bacterial immunity, but it was not until 2007 when it was demonstrated that the CRISPR/Cas system provides resistance against specific phages in the bacterial strain *Streptococcus thermophiles* [89].

**Figure 5:**
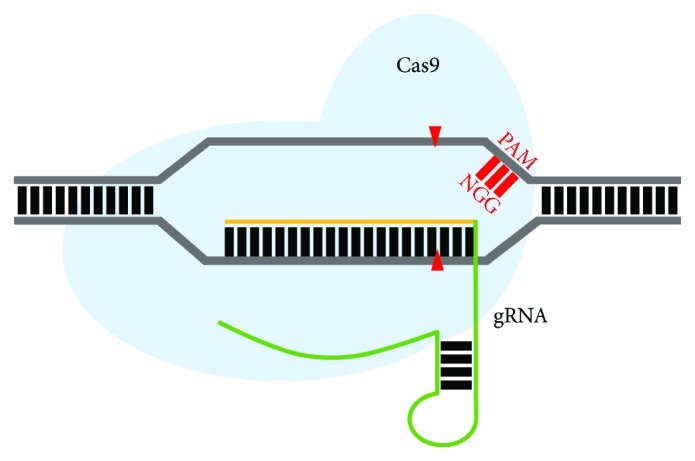
Schematic representation of the CRISPR/Cas9 system. The *Streptococcus pyogenes* Cas9 nuclease, with a “NGG” protospacer adjacent motif (PAM) sequence, has been targeted to a 20-nucleotide guide sequence in a specific region in the genome (yellow). The gRNA is complementary to the non-PAM strand. The green line represents the gRNA scaffold, which complexes with the Cas9 nuclease (light blue) and directs it to the desired site to induce a DSB (red arrowheads) in the DNA. Cas9 mediates the DSB 3 bp upstream of the PAM sequence.

CRISPR as a genome editing-system was first described in 2012 [90]. Jinek and colleagues found that the CRISPR/Cas system of *Streptomyces pyogenes* (spCas9) was capable of inducing a DSB when two RNA molecules were present, a CRISPR RNA (crRNA) and a *trans*-activating RNA (tracrRNA). In addition, the authors showed that the fusion of the crRNA and tracrRNA produces a single-guide RNA (gRNA), which is equally effective in binding to target DNA. At the 5′ end of this fused gRNA, 20 nucleotides can be customized to target specific sequences, becoming the first requirement for site-specific genome editing using CRISPR technology [90]. A second requirement for precise genome editing is found at the 3′ end directly downstream of the cleavage site, where the protospacer adjacent motif (PAM), a three-nucleotide sequence (NGG in the case of SpCas9), is an absolute requirement for Cas9 recognition (Figure 5) [91]. The combination of both, the gRNA and the PAM sequence, allows target-specific cleavage of the DNA triggered by the Cas9 endonuclease [92]. Not long after these developments, the system was used to provide efficient gene repair in cells and in numerous organisms [70, 93–97].

### 5.3. Developments and Advances in CRISPR/Cas Technology

Since the emergence of the CRISPR/Cas9 technology, researchers have focused on the development of more efficient Cas9-like nucleases, presenting similar on-target activity but reduced off-target activity. A limiting factor for the reduction of off-targets triggered by the Cas endonuclease is the cellular levels of Cas9 protein in the cells. It has been shown that high levels increase the likelihood of off-target cleavage, most likely due to the increase in mismatch tolerance between the gRNA and the DNA [98–100]. Successful efforts to overcome this limitation have been the delivery of Cas9 as a purified protein instead of using expression plasmids with strong promoters [101–105]. Alternatively, limiting the duration of Cas9 expression in the targeted cells has also been investigated. This approach has been successfully achieved in the retinal landscape as described below, presenting a huge advantage for future *in vivo* genome editing for eye diseases [106]. The use of two gRNA flanking the target region can also increase the on-target activity while reducing off-target events [92]. This strategy known as nickase Cas9 can be achieved by inactivation of one of the two nuclease domains of the Cas9, resulting in the cleavage of only one DNA strand. This strategy reduces the off-target DNA cleavage rate by 50- to 1500-fold as compared to a DSB performed at the same sequence [92]. Recent advances have come with the development of Cas9 mutants, which decrease nonspecific DNA interactions. Two parallel studies developed rationally altered spCas9 mutants (eSpCas9 and “high-fidelity” Cas9) by modification of different amino acids to significantly reduce off-target effects [107, 108]. While these new mutants and other recent approaches are promising, off-target activity for each gRNA should be tested carefully before use in the clinic to avoid unintended mutagenesis in other regions of the genome [109].

Research has also focused on increasing the repertoire of host sequences that can be targeted by the CRISPR/Cas system, which is dictated by the recognition of the PAM sequence by Cas9. Ideally, the PAM sequence should be within 10 bp of the target sequence; thus, in some regions of the genome, there might be paucity of PAM sequences. Cas9 proteins of various bacterial species have different PAM motif requirements [70, 110–112], which can be naturally exploited to expand the CRISPR/Cas target space and increase the repertoire of accessible therapeutic targets. The most commonly used Cas9 is the SpCas9, which recognizes a short NGG PAM sequence, allowing it to be used across many genomic regions [90, 113]. Other Cas9 proteins, such as those of *S. thermophilus* and *Neisseria meningitides*, require the PAM motifs NNAGAAW and NNNGATT, respectively. In addition, *Streptococcus aureus* Cas9 that recognizes a NNGRRN PAM motif has a dual interest as it is also useful for AAV delivery *in vivo* due to its smaller (3.1 kb versus 4.2 kb for SpCas9) size [112, 114]. Along this line, the 2.9 kb Cas9 from *Campylobacter jejuni* (CjCas9) also offers an attractive option for gene delivery purposes [115]. CjCas9 has been packaged into an AAV2/9 vector along with a gRNA-targeting *Vegf* and delivered to the retina in a mouse model of choroidal neovascularization (CNV) [115]. This opens up the possibility that this strategy could be an alternative to repeated administration of pharmacological anti-VEGF treatment for AMD. Lastly, the CRISPR-Cpf1 system identified in *Acidaminococcus* and *Lachnospiraceae* bacteria, which requires the PAM motif TTTN, has a dual advantage for genome editing because, in addition to a novel PAM sequence [116], it induces staggered cuts away from the critical seed region thus preventing NHEJ and increasing the efficiency of HDR [117].

To increase the repertoire of genomic target sequences even further, recent work has been aimed at artificially engineering SpCas9 and SaCas9 with alternative PAM recognition sites [118, 119]. SpCas9 recognizing PAM target sites NGA and NGCG are known as “VQR” and “VRER”, respectively; the modified SaCas9 known as “KKH” has a PAM recognition site NNNRRT. Since CRISPR/Cas9 technology was first used in 2012 for genome-editing purposes, significant advances have occurred to improve efficiency and specificity of the nucleases. The use and development of Cas9 nucleases with different PAM motifs may expand the use of CRISPR/Cas technology throughout the human genome.

## 6. *In Vivo* CRISPR/Cas Genome Editing

CRISPR/Cas genome editing in animal models has been useful for developing and testing possible therapeutic techniques that could represent sight-saving approaches in the future for patients. The biggest challenge researchers face is the delivery of the CRISPR system directly into the tissue or cells of interest in the retina. As mentioned above, AAV vectors are the most effective gene delivery method for a variety of retinal cells including photoreceptors and RPE [120]. However, their limited cloning capacity has not facilitated their application as a vehicle for CRISPR/Cas. CRISPR/Cas studies with AAV have been previously explored in the field of brain diseases [121] where the delivery of the SpCas9 and gRNA was divided between two vectors. Hung and colleagues applied a similar approach to the mouse retina whereby intravitreal administration of an AAV2/2 vector mediated the delivery of a CRISPR/Cas system designed to disrupt yellow fluorescent protein expression in a Thy1-YFP transgenic mouse model [122]. This resulted in an 84% reduction of YFP expression, providing for the first time proof of concept for CRISPR/Cas genome editing in the retina *in vivo*.

The use of dual AAV2/8 systems for the delivery of CRISPR-Cas9 components into the retina was also used to knock out the *Nrl* (neural retina-specific leucine zipper) gene in postmitotic photoreceptors. Subretinal injection of the dual AAV system prevented cone degeneration and restored the survival of rod photoreceptors in three different genetic mouse models of retinal degeneration (*Rho^−/−^* mice, *Nrl-L-EGFP/Rd10* mice, and in *RHO* P347S transgenic mice) [123]. Similarly, subretinal injection of a dual AAV2/5 CRISPR/Cas9 system in mice deleted the wild-type mouse intron 25 of the causative LCA10 gene *CEP290* [106]. This intron is homologous to the human intron 26 that houses a variant, which is the most prevalent recurrent causative mutation of LCA10 [124, 125]. Hence, this *in vivo* study is a proof of concept for the potential treatment of patients by ablation of the intronic variant [106]. In addition, the authors developed a self-limiting CRISPR/Cas9 system by incorporating recognition sites for the gRNAs into the SpCas9 plasmid, limiting the expression time of the Cas9. This self-limiting Cas9 approach lowers the chance of undesirable off-target events, potential toxicity, and SpCas9-specific cellular immune response [126].

The use of purified Cas9 ribonucleoproteins (RNP) has also been studied in the retina as an alternative delivery approach to AAV. This method reduces the time of Cas9 exposure potentially reducing off-targets, as the Cas9 RNP complex is degraded in the cell 24 h after delivery [102, 103]. Subretinal delivery of Cas9 RNP-targeting *Vegf* in a mouse model of CNV significantly reduced expression [105], thus providing preliminary evidence that this method could be used for an *in vivo* treatment of patients with AMD and more importantly expanding the possibilities for the treatment of retinal dystrophies using purified Cas9 proteins delivered directly into the retina. Further studies are needed in order to determine if the *in vivo* delivery of Cas9 RNP into the retinal cells is as efficient as viral vector-mediated delivery by subretinal injections.

These above *in vivo* studies used CRISPR/Cas9 technology to mediate NHEJ, which results in INDELs and gene inactivation. A major problem that still remains to be addressed is how to achieve effective and accurate genome editing in the retina, as photoreceptors are postmitotic cells and largely lack HDR repair mechanisms. Suzuki and colleagues developed a novel strategy called homology-independent targeted integration (HITI), which allows for targeted NHEJ knock-in in nondividing cells, such as the photoreceptors [127]. After subretinal injection of the AAV2/8- or 2/9-vehiculed HITI system in a rat model of RP, correct knock-in preserved the thickness of the ONL and improved visual function. Therefore, this approach is a highly promising solution for postmitotic neurons, as it relies on the NHEJ mechanism, as opposed to HDR, for functional integration of a desired DNA sequence.

The use of CRISPR/Cas9 system and HDR in preclinical animal models has also been performed. Wu and colleagues used this technique to determine the causative variant for the RP phenotype found in the “rodless” (rd1) mouse model. The *rd1* mice carry two homozygous variants in *Pde6b*: a nonsense mutation (Y347X) in exon 7 and a murine leukemia virus insertion in intron 1. Following CRISPR-mediated correction of the nonsense variant, the retinal phenotype of the treated mice was restored demonstrating that the Y347X mutation was pathogenic [128]. Similarly, the pathogenicity of a novel missense variant in *REEP6*-causing RP, was proven by generating a mouse knock-in model of *Reep6* using CRISPR/Cas9 technology [129].

One of the biggest challenges before the CRISPR revolution was the treatment of autosomal dominant conditions, in which specific inactivation of the mutant allele is required to restore the phenotype. The treatment of these disorders was previously considered as complicated, as gene augmentation approaches did not directly target the pathogenic gene. The development of CRISPR technology has now changed the landscape of dominant disorders. One promising therapeutic approach is to decrease gene transcription through a strategy known as CRISPR interference (CRISPRi) [130]. In this strategy, Cas9 lacks nuclease activity, known as dead Cas9 (dCas9). Blockage of the transcriptional machinery occurs when dCas9 is coupled with a sequence-specific gRNA, preventing the RNA polymerase and transcription factors from transcribing genes. This strategy has been successfully achieved in eukaryotes and human cells [131–133]. Currently, this approach has not been applied to retinal dystrophies, but it carries a great potential due to the minimal off-target effects, which is an improvement to previous strategies involving RNA interference [134, 135].

Ablation of the mutant allele using CRISPR/Cas9 technology is another strategy that has been used in dominant forms for RP due to mutations in the gene encoding Rhodopsin *(RHO)*. Bakondi and colleagues targeted an allele-specific PAM sequence present only in the *Rho^S334^* mutant allele of an RP mouse model. Following subretinal administration and electroporation of the CRISPR components, the photoreceptor phenotype was rescued and visual acuity increased by 53% [136]. Similarly, Latella et al. performed a targeted knockout of a patient-derived mutant *RHO* P23H minigene in a transgenic mouse model. Subretinal electroporation of Cas9 and two gRNA targeting the 5′ and the 3′ regions of exon 1 resulted in reduced expression of the *RHO* gene [137]. These studies carry huge promise for the use of CRISPR/Cas systems to inactivate autosomal dominant pathogenic alleles in humans.

The rapid development of these technologies and the success achieved by proof-of-concept studies *in vivo* are speeding up the clinical translation of CRISPR technology. There is currently no CRISPR-based clinical trial for eye disease. Nonetheless, this may soon change as EDITAS medicine appears dedicated to bringing the aforementioned intron 26 skipping approach for *CEP290* to LCA10 patients (https://www.allergan.com/news/news/thomson-reuters/allergan-and-editas-medicine-enter-into-strategic).

## 7. Ex Vivo Gene Correction and Cell-Based Therapy

While gene-based therapies may halt or at least slow down the progression of the disease by targeting dysfunctional cells, another promising approach in treating retinal dystrophies is stem cell-derived retinal cell transplantation. The retina develops from the neuroectoderm, thus, like any other CNS tissue, presents a low regeneration potential. Therefore, IRDs caused by degeneration or loss of photoreceptors could potentially benefit from cell-based therapies, which would restore a functional retina and reverse the ocular condition.

The first evidence showing functional photoreceptor replacement was achieved when freshly dissociated rod photoreceptors were transplanted into the subretinal space [138]. However, the number of transplanted cells could not be increased *in vitro* due to their postmitotic state. Thus, there was a need to increase the number of photoreceptors for efficient transplantation into the donor retina. Lamba and colleagues showed that human embryonic stem cells (ESCs) can be directed to a retinal cell fate and differentiated into retinal precursors [139]. The transplantation of these ESC-derived photoreceptors precursors into the subretinal space of an LCA mouse model resulted in restoration of the light response, establishing ESCs as a source for photoreceptor replacement [140]. ESCs present a high proliferative, self-renewal, and differentiation potential, which makes them an ideal tool to study human diseases *in vitro*.

However, the use of ESCs is associated with controversial and ethical considerations, thus severely impeding major progress towards exploiting their full potential. Takahashi et al. performed groundbreaking work in 2007, which overcame the major limitations associated with the use of human ESCs. Takahashi et al. demonstrated that it is possible to generate induced pluripotent stem cells (iPSCs) from adult human fibroblasts by a reprograming process, which involves expression of four transcription factors that revert the somatic cells to a pluripotent state [141]. These cells have the potential to replace patient's tissue and represent a large source of cells for the study of human disease [142, 143]. In addition, iPSC-derived cells have two major advantages in terms of cell transplantation: they avoid the ethical issues associated with the use of embryonic or fetal tissue and they offer the possibility of autologous transplantation avoiding risks of immune rejection.

Both ESCs and iPSCs have been used extensively in the area of stem cell-derived photoreceptor generation and transplantation. Sasai and colleagues revolutionized this field by showing that it is possible to mimic optic morphogenesis in 3D culture using murine [144] and human [145] ESCs and thus obtain a large source of appropriate-staged photoreceptor precursors. It was subsequently shown that, if present in sufficient numbers, both ESC-derived and donor photoreceptor precursors could restore visual function in preclinical retinal models [140, 146–149]. In addition, it was demonstrated that photoreceptor precursors [150–152] as well as functional [153] photoreceptors could also be obtained from iPSCs. Moreover, iPSC-derived photoreceptor precursors were transplantable and could also restore vision in preclinical models [154]. Human ESCs and iPSC will continue to have a huge impact on the study and the treatment of human eye disease, as more optimal and standardized differentiation protocols continue to be developed.

The coupling of iPSC and CRISPR/Cas genome-editing technologies to repair patient-specific mutations brings us to a new era of precise and personalized medicine for patients. Advances have already been made for the CRISPR/Cas-mediated correction of pathogenic mutations causing retinal dystrophies in patient's iPSCs. Bassuk and colleagues were the first to demonstrate the potential of this approach by correcting a missense mutation in *RPGR* responsible for X-linked RP [155]. Burnight and colleagues performed proof-of-concept studies for the correction of an exonic, deep intronic, and dominant gain of function variants: targeting an Alu insertion in exon 9 of *MAK* restored the retinal transcript and protein, NHEJ corrected a cryptic splice variant in *CEP290*-causing LCA10, and mutant allele-specific targeting invalidated the dominant Pro23His mutation in the *RHO* gene [156]. Further upstream, the most prevalent c.2299delG mutation in the *USH2A* gene, responsible for Usher syndrome type 2, was corrected in patient's fibroblasts using CRISPR/Cas9 and HDR [157]. These proof-of-concept studies support the development of personalized iPSC-based transplantation therapies for retinal disease. On a different note, CRISPR/Cas technology in iPSCs has been used for fluorescent reporter gene knock-in at the termination codon of the cone-rod homeobox (*Crx*) gene, a photoreceptor-specific transcription factor gene. This allows the real-time monitoring of photoreceptor differentiation [158], demonstrating the interest of this technology also for fundamental research.

Following on from the big and promising advances, which demonstrated that stem cell-derived photoreceptor transplantation can restore rod- and cone-mediated vision, recent studies demonstrated that these transplanted cells do not integrate into nondegenerative host retinas. Instead, postmitotic donor and host photoreceptors engage in the transfer of cellular material, such as RNA and proteins including Rhodopsin [149, 159–161]. The visual improvements observed after stem cell-derived photoreceptor transplantation were hypothesized to be the result of endogenous photoreceptors supplemented by donor cell-derived proteins. More recently, it was shown that both cell integration and cytoplasmic transfer can take place in degenerative hosts and that the relative contributions would depend on the local host environment [162]. Elucidation of the underlying mechanisms of this cellular material transfer could lead to novel therapeutic approaches in introducing functional proteins into dysfunctional photoreceptors as an alternative to gene replacement. In particular, it opens up the attractive possibility that Cas9 could be delivered as a purified protein for genome editing of viable photoreceptors.

The use of stem cell-derived photoreceptors is a powerful tool for the understanding of human retinal development and disease modeling and underlies a great potential for developing cell transplantation therapies. Such therapies are already underway in the clinic using hESC- [163–165] or hiPSC-derived [166] RPE. Initially, hESC-derived RPE was subretinally administered into AMD and Stargardt patients as dissociated cells. These cells safely persisted over time in the host retina and stably rescued visual acuity in a subset of patients [164]. Just recently, an RPE patch comprising a fully differentiated hESC-derived RPE monolayer on a coated, synthetic basement membrane was transplanted into AMD patients [165]. A one-year follow-up showed persistence of the sheet, which was associated with increased visual acuity and reading speed. It remains to be seen if these improvements will be stable over time. Lastly, the first ever, autologous transplantation for the retina was performed using a free hiPSC-derived RPE monolayer [166]. A one-year follow-up showed that the transplantation was safe and no immune response was provoked even in the absence of immunosuppression. This provides hope for the future autologous transplantation of genome-edited retinal cells in patients. Nonetheless, further work is required to establish robust and reproducible protocols for the generation of iPSC-derived photoreceptors. In addition, if such cells are transplanted following gene mutation repair, stringent quality control of the iPSCs before and after gene correction is extremely important. Furthermore, a detailed screening for possible off-target effects triggered by CRISPR/Cas has to be performed prior to transplantation into the diseased host retina.

## 8. Future Challenges and Perspectives

The eye, more specifically the posterior retina, has proven to be a powerful model for the development of pioneer therapies, which could later be applied to other parts of the CNS. Despite the current success achieved by researchers and the relative ease and precise manipulation of the genome using the CRISPR/Cas system, improvements are being made. These are focused on the development of more efficient delivery methods, the identification and understanding of the off-target events, and increasing the efficiency of mutation correction. All these matters should be carefully addressed before this strategy can be safely applied in the clinic.

Potential delivery methods of the CRISPR/Cas components can be diverse. For an *in vivo* application, the ideal vehicle would be an AAV vector. The limitation of this method, in addition to size restrictions, is the constitutive expression of the Cas9 protein in the host organism, which increases the risk of unwanted off-target events in the genome [98–100]. The use of Cas9 RNP has been shown to be effective *in vivo* for reducing off-target events [101, 102, 167], although to our knowledge there has not been a study directly comparing the off-target effects of a given gRNA by AAV or RNP delivery. Thus, future research is needed in order to elucidate the most effective way, with high on-target activity and null off-target activity, to deliver CRISPR/Cas components *in vivo*.

A variety of methods aimed at testing for off-target mutations have been developed [168–170]. These methods are based on algorithms to computationally test homologous regions in the genome. However, currently, there is no gold standard, and it is not yet clear if Cas9 has the potential to alter other nonhomologous regions in the genome. Some studies have performed whole exome sequencing (WES) in CRISPR-treated cells and organisms [171, 172], providing an accurate and comprehensive way of testing off-target mutations. Such approaches should be taken into consideration following ex vivo gene correction in view of future transplantation into the patient.

In addition to improving the understanding of the off-target effects created by Cas9, much effort has focused on developing methods to enhance genome-editing efficiency. In cases where gene correction is required, the HDR repair pathway is needed, and this is incompatible with postmitotic photoreceptor targets. Exciting new developments in HDR-independent base-editing strategies have shown promise for gene correction in postmitotic cells. In these cases, Cas9 is fused to a cytidine deaminase to create a base-editor tool at the specific genome target [173, 174], thus circumventing the need for cell division. In addition, as mentioned above, the HITI approach also carries a great promise for precise gene correction in postmitotic cells by using the NHEJ pathway [127].

Overall, the future looks bright for the use of CRISPR/Cas genome editing in ophthalmology, and it is likely that the studies presented here are just the beginning of what is to come.
